# Blunt Traumatic Retropharyngeal Hematoma with Respiratory Symptoms: A Systematic Review of Reported Cases

**DOI:** 10.1155/2021/5158403

**Published:** 2021-10-07

**Authors:** Yu-Ling Tsao, Chien-Chin Hsu, Kuo-Tai Chen

**Affiliations:** ^1^Emergency Department, Lienchiang County Hospital, Lienchiang County, Taiwan; ^2^Emergency Department, Chi-Mei Medical Center, Tainan, Taiwan; ^3^Department of Biotechnology, Southern Tainan University of Technology, Tainan, Taiwan

## Abstract

**Background:**

In patients with blunt trauma, particularly geriatric patients and those with minor trauma, an insidious retropharyngeal hematoma (RH) may deteriorate and have lethal consequences. We review the relevant literature to elucidate the clinical characteristics, treatment, complications, and outcomes of blunt traumatic RH with respiratory symptoms. *Data Resources*. We reviewed 57 case reports and added one case from our hospital for data analysis. A total of 68 cases were included in this review.

**Results:**

The ages of patients ranged from 13 to 94 years, and geriatric patients (age >66 years) constituted 61.2% of the reviewed patients. Falls (54.4%) and traffic accidents (35.3%) were the major trauma mechanisms. Most patients' symptoms developed within 24 hours of blunt trauma (95.2%), and 73.5% of patients with RH had at least one associated injury. Many patients underwent conservative treatment for RH (63.2%). Surgical treatment (23.5%) and transarterial embolization (8.8%) were used to control retropharyngeal hemorrhage. Twelve patients died; RH and cervical spinal injury were the direct causes of death in 5 patients, whereas the other 7 patients died because of cardiac, pulmonary, or gastrointestinal causes or withdrawal of life support.

**Conclusions:**

Geriatric patients constituted the largest proportion of patients with RH, and minor trauma was adequate to result in RH in elderly people. The cornerstone of RH management is airway management. Surgery and transarterial embolization are commonly used to control active bleeding in patients with RH. The long-term outcome depends on patients' associated injuries and in-hospital complications.

## 1. Introduction

Retropharyngeal hematoma (RH) is a potentially life-threatening disease because the expanding hematoma may progress to completely obstruct the airway. RH has diverse etiologies, including anticoagulant use, hematologic illness, iatrogenic procedure, soft tissue infection, penetrating injury, and idiopathic [1–8]. Except in cases of spontaneous RH, the underlying conditions and preceding events usually remind healthcare workers that RH is the cause of a patient's respiratory symptoms. Nevertheless, in patients with blunt trauma, particularly older patients and those with minor trauma, insidious RH may deteriorate and be lethal.

RHs are not uncommonly discovered in trauma patients, especially patients with cervical injuries. Penning had reported that 60% of patients with cervical injury had widening of the prevertebral space [9]. However, airway obstruction due to RH occurs in only 1.2% of patients [10]. Urgent airway management is not indicated in asymptomatic patients. For emergency physicians and trauma surgeons, knowing the typical history and proper treatment, including airway management, for traumatic RH is essential. We could not find a clinical study concerning blunt traumatic RH with respiratory symptoms because the few cases are dispersed among various countries and hospitals. Therefore, we collected and reviewed every reported case of traumatic RH with respiratory symptoms. The aim of this review is to elucidate the clinical characteristics, treatment, complications, and outcomes of patients with this condition.

## 2. Materials and Methods

The approval of an institutional review board was not required because this study did not involve human subjects or chart review.

We performed an electronic search of the literature published between 1940 and 2018 by using the following keywords: “retropharyngeal hematoma” and “prevertebral hematoma.” The databases searched were PubMed and Google Scholar. The references of all articles were manually searched to identify articles missed in the electronic search. The search was international, and no language limitation was imposed. Studies including patients with penetrating injury, anticoagulant use, coagulopathy, neck-infection-related hematoma, RH without any respiratory symptoms, and spontaneous RH were excluded. Only studies recruiting patients with blunt traumatic RH and respiratory symptoms were included.

**Figure 1:**
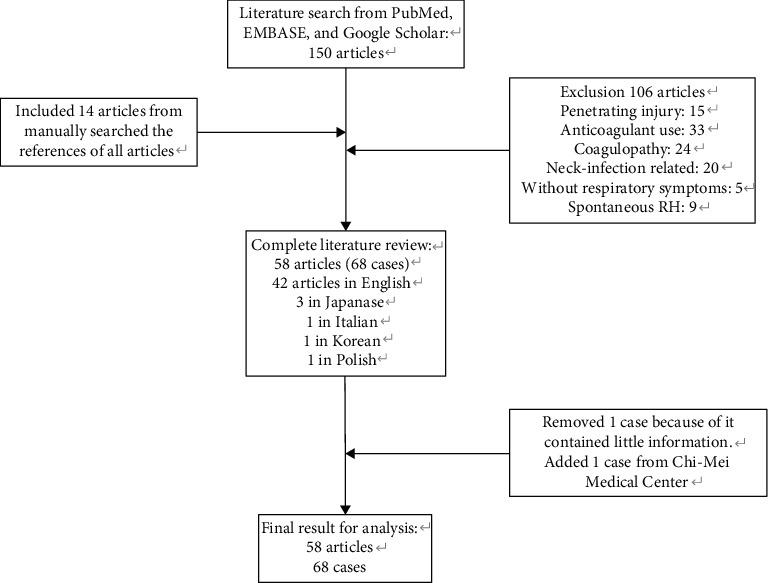
The flowchart of literature research.

Fifty-eight articles with 68 cases—including 42 articles in English, 3 in Japanese, 1 in Italian, 1 in Korean, and 1 in Polish—met our search criteria. We excluded the first case of a collected article because it contained little clinical information and was useless for further analysis [11]. Subsequently, we added one case from Chi-Mei Medical Center for data analysis. A total of 68 cases were included in this reviewed article (Table 1). The flowchart of literature research is shown in Figure 1. Some patient characteristics were not revealed in all the papers; therefore, the sum for each item may not be 68.

## 3. Results

### 3.1. Demographics, Clinical Presentation, and Associated Injuries

**Figure 2:**
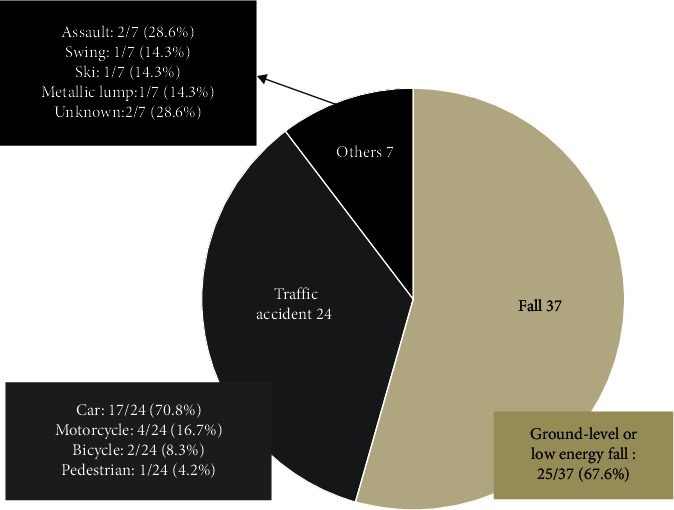
Mechanisms of trauma.

**Figure 3:**
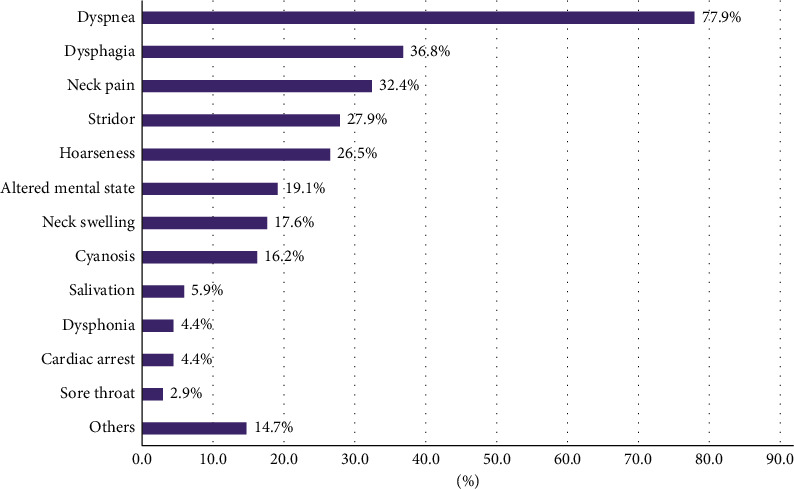
Common symptoms for patients with retropharyngeal hematoma and respiratory symptoms.

The ages of patients ranged from 13 to 94 years (median (interquartile range): 72 (50–82) years). Geriatric patients (age >64 years) constituted 61.2% of the reviewed patients. In accordance with the distributions of most reports of trauma, male predominance was discovered in patients with blunt traumatic RH (male vs. female: 76.5% vs. 23.5%). Falls (54.4%), particularly ground-level or low-energy (fall from height <2 m) falls, and traffic accidents (35.3%) were the major causes of trauma (Figure 2). Common symptoms for patients with RH and respiratory symptoms were dyspnea (77.9%), dysphagia (36.8%), neck pain (32.4%), stridor (27.9%), hoarseness (26.5%), altered mental state (19.1%), neck swelling (17.6%), and cyanosis (16.2%) (Figure 3). Most patients' symptoms developed within 24 hours of the blunt trauma (95.2%).

**Figure 4:**
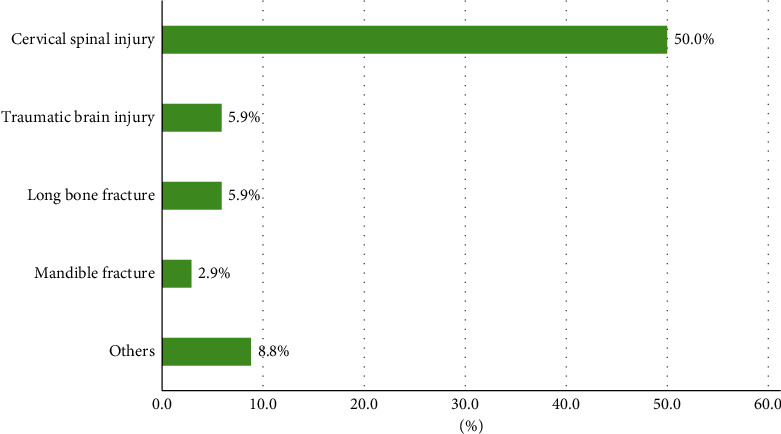
Associated injuries. ^*∗*^Others included thoracic aortic dissection, lung contusion, brachial plexus injury, cranial nerve VI palsy, skull fracture, and rib fracture.

Nearly three-quarters of patients with RH had at least one associated injury (73.5%). The common associated injuries were cervical spinal injury (50.0%), traumatic brain injury (5.9%), long bone fracture (5.9%), and mandibular fracture (2.9%) (Figure 4). Roentgenography of the neck or cervical spine (77.9%) was the initial diagnostic tool in most cases, and only 1 in 53 images failed to show enlargement of the prevertebral space. Other methods of diagnosing RH were computed tomography of the neck (67.6%), a fiberscope (42.6%), angiography (14.7%), magnetic resonance imaging of the neck (13.2%), and autopsy (2.9%).

### 3.2. Airway Management, Treatment, and Prognosis

**Figure 5:**
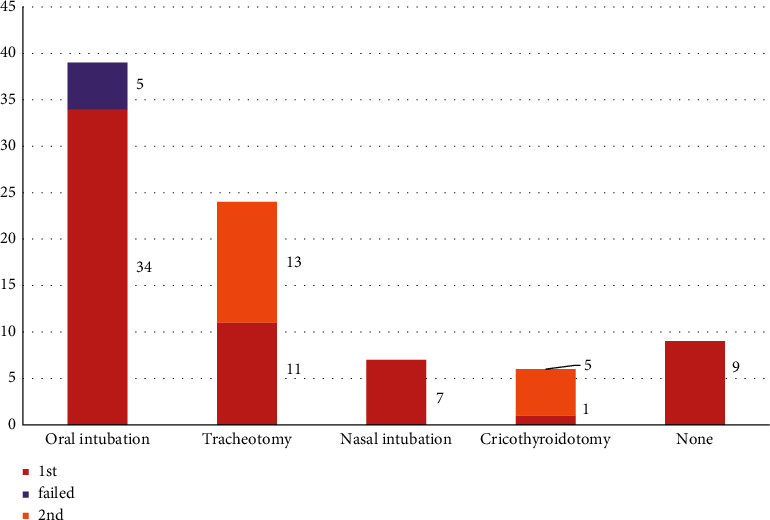
Airway management of patients. The brick red bars indicate the first successful attempt to secure the patient's airway. The purple bar indicates 5 failed attempts at oral intubation. The yellow bars indicate the rescue method or requirement for airway management.

Oral tracheal intubation remains the most common method of establishing a secure airway. Tracheostomy, nasal tracheal intubation, and cricothyroidotomy are also useful approaches in patients with a threatened airway. Notably, in the reviewed cases, surgical airways often served as a rescue method when tracheal intubation failed. Death occurred before airway management could be implemented in 2 patients, and 7 patients did not require an artificial airway (Figure 5).

**Figure 6:**
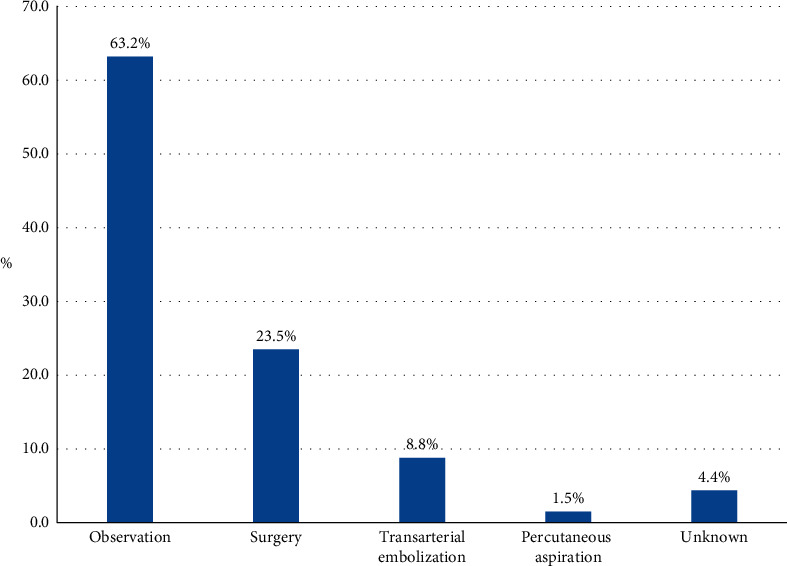
Treatment for retropharyngeal hematoma. One patient underwent transarterial embolization first. Once the active bleeding had ceased, surgical evacuation of the hematoma was performed. Thus, 69 treatments were performed in total.

Most patients underwent conservative treatment (63.2%) for RH. Surgical treatment (23.5%) and transarterial embolization (8.8%) were used to control retropharyngeal hemorrhage. One patient underwent transarterial embolization followed by surgical drainage of the residual hematoma. Percutaneous aspiration was conducted in one patient (Figure 6).

The prognosis of 2 patients is unknown. Of the remaining 66 patients, 12 died, which yielded a mortality rate of 18.2% (12/66). RH and accompanying cervical spinal injury were the direct causes of death in 5 patients. Another 7 patients expired from cardiac, pulmonary, or gastrointestinal causes or withdrawal of life support. Nine patients had undergone cardiopulmonary resuscitation before their hospitalization or in an emergency department, and acute airway obstruction was considered the etiology of cardiac arrest in these patients. Among these 9 patients, 4 died, and 3 survived; the outcome for 2 patients is unknown. The mortality considerably increased to 57.1% in patients who had experienced a cardiac arrest event during treatment.

### 3.3. Pre- and Postintervention Salient Image Features

**Figure 7:**
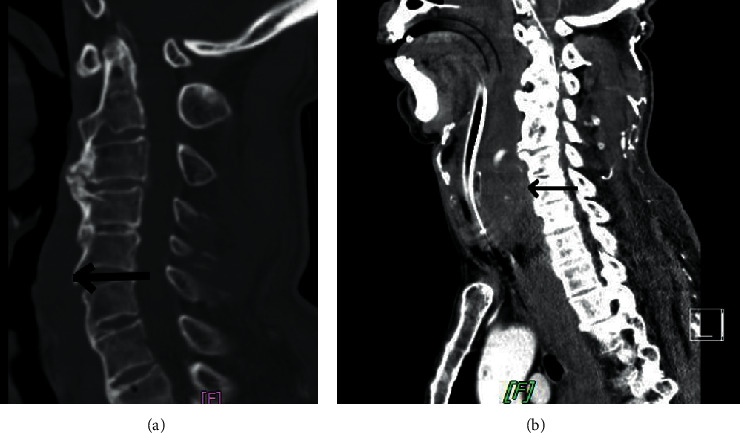
The dynamic changes from an initial computed tomographic scan of the cervical spine (a) to a later computed tomographic scan of the neck (b). Retropharyngeal hematoma (arrow) expanded significantly 4 hours after injury. In Figure 7(b), the patient had undergone tracheal intubation, and extravasation of contrast media in the hematoma indicated an active bleeder.

Taken from the images of the patient in Chi-Mei Medical Center, we presented an initial computed tomographic scan of the cervical spine and a later computed tomographic scan of the neck. Retropharyngeal hematoma expanded significantly 4 hours after injury (Figure 7).

## 4. Discussion

Several mechanisms have been proposed for the development of blunt traumatic RH. Whiplash injury, which is a hyperextension injury of the cervical vertebrae, can result in tearing of the longus colli muscle or anterior longitudinal ligament [12, 13]. Additionally, fracture of the cervical vertebrae may damage the surrounding soft tissue and small branches of vertebral arteries [14]. We discovered that 68% of patients with RH had an associated cervical spinal injury, including cervical spinal fracture or dislocation and ligament injury, supporting this hypothesis. Nérot et al. reported a case of esophageal perforation after fracture of the cervical spine. Impingement of the esophagus against an exostosis in an osteoarthritis-stiffened spine was the proposed mechanism [15]. In our reported case, the elderly man had clear osteophytes on his cervical vertebrae in roentgenographic imaging of his neck, which boosted the suspicion of this mechanism.

Elderly people are vulnerable to falling and usually sustain severe injury when they do fall [16, 17]. In this review, we discovered that the median age of all reported cases was 72 years, and geriatric patients constituted the largest proportion of all patients. Additionally, most falling accidents were ground-level and low-energy falls. In combination with the reviewed literature, evidence indicates that a high index of suspicion for RH should be applied to geriatric patients with trauma, even for patients who have sustained only minor trauma.

The respiratory symptoms of most patients included in this study occurred within 24 hours of injury. Delayed presentation of symptoms was rare. Therefore, we recommend that trauma surgeons and emergency physicians observe patients closely and inform patients and their caregivers that acute respiratory symptoms might occur within 24 hours after the injury. If any symptoms of respiratory compromised arise, urgent evaluation for RH and preparation to secure the patient's airway are crucial means of care for these patients.

The earliest diagnostic tool in most patients was roentgenography of the neck or cervical spine, and 98% of the plain films showed enlargement of the prevertebral space, which is in accordance with a previous study reporting that RH is a common finding in patients with cervical spinal injury [9]. Nevertheless, the presence of RH does not guarantee the existence of respiratory symptoms. Many patients with RH are asymptomatic. Additionally, the extent of RH shown on the plain film does not disclose the probability of subsequent airway obstruction. Trauma surgeons or emergency physicians used computed tomography of the neck, a fiberscope, and magnetic resonance imaging of the neck to assess patients with RH that could compromise their airway and the resolution of RH. If immediate securing of a patent's airway is not indicated, we advocate routine utilization of a fiberscope in these patients. A fiberscope can be employed to identify swelling of the posterior pharyngeal wall, accurately evaluate the patency of the airway, and determine the optimal means of establishing an artificial airway. Besides, keeping the affected patient in an upright position facilitates breathing, and the examining physician can repeat the procedure to evaluate the progression of RH.

During evaluation of RH and airway patency, trauma surgeons and emergency physicians should also be concerned about the injuries associated with RH. We discovered that many of the associated injuries presented on head and neck regions in our enrolled patients. Most of these injuries could be observed in roentgenography, computed tomography, and magnetic resonance images. The management and outcome of patients with RH were influenced by these associated injuries. The decision of how to secure the airway is conceivably affected by the existence of cervical spinal injuries. Concomitant traumatic brain injury might alter the prognosis of patients, and mandible fracture may impede tracheal intubation.

For patients with traumatic RH, the major cause of death is RH-induced acute airway obstruction. Accordingly, the cornerstone of initial management for patients with RH is securing the patent's airway. In most circumstances, the patient's acute airway obstruction occurs abruptly. Therefore, oral intubation remains the intuitive option for most doctors. However, performing oral intubation in a patient whose airway is distorted by RH may be difficult, and cervical immobilization may be required during the procedure. In selected cases, if the doctor is familiar with the operation of a fiberscope, this is a safe and practical method of scope-guided nasal intubation in patients with RH. Tracheostomy is also suitable because of the concern for concomitant cervical spinal injury and mandible fracture. Both tracheostomy and cricothyroidotomy can serve as rescue management approaches if the initial intubation attempt fails.

For traumatic RH, observation and conservative management are the most common treatment, which is in accordance with the trend for nonoperative management of traumatic injuries [18]. The duration of observation varied in the reviewed reports, ranging from 4 days to 2 weeks [19, 20]. We cannot suggest a practical observational period for the treatment of patients with RH. Surgery is a traditional means of identifying the bleeder, stopping the bleeding, and evacuating the hematoma. However, operating on a patient with concomitant head and neck injuries may be difficult. Because of advances in imaging techniques and endovascular procedures, transarterial embolization has become an adjunct for nonoperative treatment^21^. We discovered that more patients have undergone transarterial embolization to stop active bleeding in RH in recent years [13]. The requirement for surgical treatment for RH has decreased. If the bleeder can be identified and stopped, the residual hematoma should absorb uneventfully. We did not discover any report that mentions complications related to residual RH.

The direct cause of death resulting from RH is unanticipated airway obstruction. Inappropriate or delayed airway management leads to a high mortality rate in patients experiencing cardiac arrest. If patients survive the initial life-threatening period, mortality is not related to RH. The long-term outcome depends on patients' associated injuries and the in-hospital complications of geriatric patients.

## 5. Conclusion

Geriatric patients constituted the largest proportion of patients with RH, and minor trauma was discovered to be adequate to result in RH in elderly people. Most respiratory symptoms occurred within 24 hours of injury. Most patients with RH had at least one associated injury, which often presented in the head or neck. The cornerstone of management for patients with RH is airway management. Surgery and transarterial embolization are commonly used to control active bleeding in patients with RH. The residual hematoma should absorb uneventfully. The long-term outcome depends on patients' associated injuries and in-hospital complications.

## Figures and Tables

**Table 1:** Reviewed articles and characteristics of the included patients.

Author, year	Country	Age	Sex	Type of injury	Time from injury to symptoms (day)	Symptoms of RH	Associated injuries	Methods of airway management	Treatment modality	Hospital stay	Cause of death
Logan GE, 1962^12^	US	43	M	Fall	1	Neck swelling, dysphagia, dyspnea	C5-6 fr	Trach	Observe		0
Sandor F, 1964^13^	UK	73	M	Fall	Unknown	Neck pain, dyspnea, dysphagia	C6 fr	None	Observe	5	RH
Miller CH, 1970^11^	US	85	M	Fall	Unknown	Dyspnea, superior vena cava syndrome	Thoracic aortic dissection	OTI/Trach	Observe	?	GI bleeding
Stein H, 1970^14^	US	46	M	Assault	1	Dysphagia	Nil	None	Percutaneous aspiration	6	0
Howcroft AJ, 1977^15^	UK	73	M	MVA	1	Neck pain, neck swelling, dyspnea, cyanosis, drooling	C5 fr	Trach	Surgery	14	0
O'Neill JV, 1977^16^	US	80	F	Fall	1	Dysphagia, dyspnea, hoarseness, salivation	Nil	Trach	Observe	8	Cardiac cause
Wong YK, 1978^17^	Canada	75	M	Fall	1	Dyspnea, stridor, tachypnea	C5 fr	OTI	Observe	?	0
Smally AJ, 1981^18^	US	75	F	MVA	1	Neck pain, stridor, hoarseness, cyanosis	Nil	OTI/Trach	Unknown	0	0
Irvine GH, 1984^19^	UK	62	M	Assault	1	Dyspnea, hoarseness, orthopnea, cyanosis	Nil	Trach	Observe	?	0
Coleman JA Jr, 1986 (1)^20^	US	69	M	Fall	1	Hoarseness, stridor	C6 fr, quadriplegia	Trach	Observe	14	Cervical cord injury
Coleman JA Jr, 1986 (2)	US	82	M	Fall	1	Stridor, neck pain	C2 fr	NTI/Trach	Observe	?	0
Smith JP, 1988^21^	US	77	M	Fall	1	Neck pain, stridor, dyspnea, dysphagia	Nil	Trach	Observe	?	0
Myssiorek D, 1989^22^	US	80	M	MVA	1	Dysphagia, hoarseness, dyspnea	Nil	OTI	Surgery	10	0
Biby L, 1990^23^	US	27	F	MVA	1	Neck pain, dysphagia, trismus	Cranial nerve VI palsy	NTI	Observe	?	0
McLauchlin CJ, 1991^24^	UK	76	M	Fall	1	Dyspnea, stridor, AMS	Nil	OTI failed/Cric	Observe	?	0
Haraguchi K, 1991^25^	Japan	72	M	Fall	1	Dyspnea	Nil	Trach	Observe	?	0
Kuhn JE, 1991 (1)^10^	US	22	M	MVA	1	Dyspnea, salivation, stridor	Mandible fr, C5, 6 fr-subluxation	OTI failed/Cric	Observe	28	Brain death
Kuhn JE, 1991 (2)	US	70	M	MVA	1	Dyspnea, cyanosis, AMS	Mandible fr, C2, 3 fr-subluxation, C7 fr	OTI	Observe	?	0
Kuhn JE, 1991 (3)	US	58	M	Fall	1	Stridor, salivation	C1-3 fr	Trach	Observe	?	0
Kuhn JE, 1991 (4)	US	82	M	Fall	1	Dyspnea, stridor	Clavicle fr	OTI/Cric	Observe	9	0
Kuhn JE, 1991 (5)	US	75	M	MVA	1	Dyspnea	C4, 5 fr	OTI	Observe	?	PN
Kuhn JE, 1991 (6)	US	92	M	Fall	1	Dyspnea	C5, 6 fr, paraplegia	OTI failed/Trach	Observe	?	WDLS
Kuhn JE, 1991 (7)	US	83	M	MVA	1	Cardiac arrest	C4, 5 fr-dislocation, quadriplegia	OTI failed/Cric	Observe	3	WDLS
Daniello NJ, 1994^26^	US	57	F	Fall	1	Dyspnea, dysphagia	Nil	OTI	Surgery	7	0
Corbanese U, 1995^27^	Italy	24	M	MVA	1	AMS, dyspnea, cyanosis, dysphagia, neck pain, hoarseness	SAH, fractured leg	OTI/Trach	Observe		0
Mitchell RO, 1995^28^	US	28	F	MVA	1	Hoarseness, stridor, neck pain, agitation	Nil	NTI/Trach	Observe	?	0
Shaw CB, 1995^29^	US	?	M	Pedestrian struck by a car	1	Dyspnea, dysphagia	Nil	OTI/Trach	Surgery	?	0
O'Donnell JJ, 1997^30^	Ireland	19	M	MVA	1	AMS, dyspnea, cyanosis, bloody vomitus	Atlanto-occipital fr-dislocation, lung contusion	OTI	Observe	2	Cervical cord injury
Mazzon D, 1998^31^	Italy	82	M	MVA	1	AMS, cyanosis	C4, 5 fr	OTI/Trach	Observe	?	0
Cox RG, 1998^32^	Canada	13	F	Bicycle accident	1	Neck pain, stridor, dysphagia, drowsy	Skull fr	OTI	Observe	?	0
Taguchi T, 1998^33^	Japan	66	M	Fall	1	Neck swelling, dyspnea	C6 fracture	OTI	Surgery	?	0
Tsai KJ, 1999^34^	Taiwan	54	M	MVA	1	AMS, dyspnea, cyanosis, dysphagia	Nil	OTI	Observe	9	0
Vakees YS, 2000^35^	UK	88	F	Fall	1	Dyspnea, hoarseness, stridor	Nil	None	Observe	?	0
Kette F, 2000^36^	Italy	67	M	Fall	161	Dyspnea, neck pain, hoarseness	Nil	OTI	Observe	14	0
Kettani CE, 2002^37^	France	37	M	MVA	1	Dyspnea, neck pain, stridor, dysphagia	Clavicle fr, C6 fr	NTI	Observe	9	0
Velde RV, 2002^38^	Netherlands	84	F	Fall	1	Dyspnea, sore throat, neck swelling, stridor	Nil	OTI	TAE	22	0
Kim SB, 2003 (1)^39^	Korea	60	M	Fall	1	Dyspnea, neck swelling	Nil	Trach	Surgery	5	0
Kim SB, 2003 (2)	Korea	44	M	Unknown	1	Dyspnea	C4, 5 fr	OTI	Observe	16	0
Shiratori T, 2003^40^	Japan	40	M	Skiing accident	1	Dyspnea, neck pain	Nil	OTI	Observe	14	0
Kochilas X, 2004^41^	UK	53	M	Fall	1	Stridor, dysphonia, dysphagia, neck swelling	Nil	None	Observe	6	0
Suzuki T, 2004^42^	Japan	67	M	MVA	1	Neck pain, dyspnea, cyanosis, AMS	C5 fr	OTI	Observe	1	RH
Collins KA, 2005^43^	US	94	M	Fall	1	AMS, dyspnea	C5 fr	None	Nil	0	RH
Clifton R, 2005^44^	UK	66	F	MVA	1	Neck pain, dysphagia	Nil	None	Observe	?	0
Duvillard C, 2005 (1)^45^	France	40	M	Struck by a metallic lump	Unknown	Dyspnea, dysphagia	Nil	Trach	Surgery	14	0
Duvillard C, 2005 (2)	France	94	M	Fall	Unknown	Dysphagia	Nil	None	Observe	6	0
Anagnostara A, 2005^46^	Greece	58	M	MVA	1	Sore throat, hoarseness, dysphagia, dyspnea, neck bruise	Nil	None	Observe	5	0
Sheah K, 2006^47^	Singapore	90	M	Fall	Unknown	Neck swelling, stridor	Nil	OTI	TAE/surgery	10	0
Takeuchi S, 2007^48^	Japan	31	M	Motorcycle accident	1	Neck pain, dyspnea, hoarseness	Atlanto-occipital dislocation, mandible fr, SAH	OTI	Surgery	73	0
Lin JY, 2007^49^	Taiwan	50	M	Fall	1	Neck swelling, dyspnea, hoarseness	Nil	NTI/Trach	Surgery	9	0
Lazott LW, 2007^50^	US	50	M	Fall	1	Neck pain, dyspnea, hoarseness	C1 fr, brachial plexus injury	NTI	Surgery	5	0
Gotlib T, 2008^51^	Poland	85	F	Struck by a swing	1	Dysphagia, hoarseness, dyspnea	Nil	None	Observe	5	0
Tsai SH, 2008^52^	Taiwan	40	M	Fall	1	Dyspnea, dysphonia	Nil	Trach	Observe	10	0
Morita S, 2010^53^	Japan	92	M	Fall	1	Throat pain, neck pain, dyspnea	C4, 5 ALL injury	OTI	Observe	14	0
Birkholz T, 2010^54^	Germany	77	M	MVA	1	Dyspnea, cardiac arrest	C2 fr, quadriplegia, fractured leg	OTI failed/Cric	Observe	?	0
Pfeiffer J, 2011^55^	Germany	92	F	Fall	1	Dyspnea, dysphagia, throat foreign body sensation	Cervical spinal ALL injury	OTI	Surgery	8	0
Wronka KS, 2011^56^	UK	89	M	Fall	7	Dysphagia, hoarseness, dyspnea, stridor	C2 fr	OTI/Trach	Nil	?	0
Iizuka S, 2012^57^	Japan	30	F	MVA	1	Neck pain, dyspnea, stridor	Intracranial hemorrhage, C4-7 fr	OTI	Observe	22	0
Jakanani G, 2012^58^	UK	65	F	Fall	1	Dyspnea, cardiac arrest	C5 fr	OTI	TAE	?	Unknown
Cleiman P, 2012^59^	Canada	87	M	Fall	1	Dyspnea, AMS	C5 fracture	Cric	Unknown	?	Unknown
Senel AC, 2012^60^	Turkey	86	F	Fall	Unknown	Dyspnea, cyanosis, neck swelling	Nil	OTI/Trach	Observe	10	PN
Park JH, 2015 (1)^61^	Korea	51	M	Fall	1	Neck pain, dyspnea	Cervical spinal ALL injury	OTI	Surgery	7	0
Park JH, 2015 (2)	Korea	78	M	Unknown	Unknown	Dyspnea, neck swelling	RH	OTI	Surgery	8	0
Paul D, 2015^62^	India	78	M	Fall	1	Dysphagia, neck pain, dyspnea, AMS, stridor, cyanosis, hoarseness	Nil	Trach	Surgery	10	Cardiac cause
Calogero CG, 2015^63^	US	80	M	Fall	1	Dysphagia, neck swelling, hoarseness, dyspnea	Nil	OTI	TAE	?	0
Kudo S, 2017^64^	Japan	83	F	MVA	1	AMS, dyspnea, shock, neck swelling	SAH, C4, 5 dislocation	OTI	TAE	?	0
Lowe E, 2017^65^	UK	60	F	Fall	Unknown	Hoarseness, dysphagia, neck pain	Nil	NTI/Trach	Surgery	?	0
Mira MD, 2018^66^	Spain	80	M	Fall	Unknown	Dyspnea, dysphagia	C6 fr	OTI	Observe	17	0
Tsao YL, 2018	Taiwan	76	M	Bicycle accident	1	Dyspnea, increased airway secretion, AMS	C4-6 fr, rib fr	OTI	TAE	40	0

M: male; F: female; fr: fracture; C: cervical spine; RH: retropharyngeal hematoma; Trach: tracheostomy; OTI: oral tracheal intubation; GI: gastrointestinal; MVA: motor vehicle accidents; NTI: nasal tracheal intubation; Cric: cricothyroidotomy; PN: pneumonia; WDLS: withdrawal of life support; AMS: altered mental status; TAE: transcutaneous arterial embolization; SAH: subarachnoid hemorrhage; ALL: anterior longitudinal ligament. ?Each reviewed article included 1 to 7 cases.

## Data Availability

The data were taken from the review of every collected manuscript and one case from Chi-Mei Medical Center.
